# The sex‐specific association between long‐term PM2.5 exposure and incident dementia in community‐dwelling older adults in Australia

**DOI:** 10.1002/alz.71256

**Published:** 2026-03-04

**Authors:** Aoshuang Zhou, Zhen Zhou, Wenhua Yu, Tingting Ye, Alice Owen, Robyn Woods, Suzanne Orchard, Rory Wolfe, Yuming Guo, Joanne Ryan

**Affiliations:** ^1^ Biological Neuropsychiatry & Dementia Unit, School of Public Health and Preventive Medicine Monash University Melbourne Victoria Australia; ^2^ Climate, Air Quality Research Unit, School of Public Health and Preventive Medicine Monash University Melbourne Victoria Australia; ^3^ School of Public Health and Preventive Medicine Monash University Melbourne Victoria Australia

**Keywords:** air pollution, dementia risk, older adults, PM2.5, sex differences

## Abstract

**INTRODUCTION:**

Air pollution is linked to dementia, but evidence from low‐exposure settings is limited. We examined sex‐specific associations between long‐term exposure to fine particulate matter ≤2.5 µm in diameter (PM2.5) and dementia risk in older adults living in Australia.

**METHODS:**

In 16,145 dementia‐free Aspirin in Reducing Events in the Elderly (ASPREE) participants (≥70 years; median follow‐up 10.3 years), Cox models assessed associations between 1‐year mean PM2.5 (continuous and guideline‐based categories) and incident dementia, adjusting for demographic, lifestyle, environmental, and genetic factors. Subgroup analyses by sex, apolipoprotein E genotype (*APOE*), and age were conducted.

**RESULTS:**

Overall associations were null, but with a trend for increased risk at exposures >10 versus ≤5 µg/m^3^. In subgroup analyses, positive associations were observed among females, with larger effect estimates at exposure >10 µg/m^3^, whereas associations remained null among males. No differences were observed across *APOE* genotypes or age groups.

**DISCUSSION:**

Findings suggest a threshold of >10 µg/m^3^ and heightened susceptibility in females. Further research in low‐exposure settings is warranted.

## HIGHLIGHTS

- **Threshold effect**: Although not statistically significant in the overall population, exposure > 10 µg/m^3^ was associated with increased dementia risk (HR 1.50; 95% CI: 0.97 to 2.31).
- **Sex difference**: Females showed a 7% higher dementia risk per 1 µg/m^3^ increase (HR 1.07; 95% CI: 1.01 to 1.14); no association was observed in males (HR 1.00; 95% CI: 0.95 to 1.07).
- **Female vulnerability**: Females exposed to >10 µg/m^3^ had a 2.7‐fold higher risk (HR 2.71; 95% CI: 1.55 to 4.74) compared with ≤5 µg/m^3^.

## 1 BACKGROUND

As the global population ages, the burden of dementia continues to grow.^1^ There is a well‐established link between exposure to ambient air pollution, particularly fine particulate matter ≤2.5 µm in diameter (PM2.5), and increased dementia risk.^2^, ^3^, ^4^, ^5^ Importantly, unlike many individual‐level factors, air pollution is a population‐level risk factor that can be mitigated through informed policies and environmental regulations, underscoring its significance in large‐scale preventive strategies.^5^ However, current air quality guidelines vary across jurisdictions and were primarily developed to address the risks of premature mortality.^6^, ^7^ For instance, the World Health Organization (WHO) sets an annual mean PM2.5 threshold of 5 µg/m^3^, the European Union (EU) recommends 10 µg/m^3^ by 2030, and Australia follows a national standard of 8 µg/m^3^. While these values provide important benchmarks for mortality risk, it remains unclear whether they are fully applicable to dementia prevention.

PM2.5, defined as particles with an aerodynamic diameter ≤ 2.5 µm, is a major contributor to air pollution.^8^ Due to their small sizes, PM2.5 particles can penetrate deep into the lungs and trigger systemic inflammation^9^ or oxidative stress.^10^ These systemic responses may, in turn, lead to neuroinflammation, negatively affecting the central nervous system.^11^ In addition to these indirect mechanisms, PM2.5 particles may directly affect the brain by bypassing protective barriers via the olfactory bulb or by crossing the blood–brain barrier.^12^, ^13^, ^14^ Indeed, a recent systematic review^4^ of 28 studies found at least a 14% higher risk of dementia across PM2.5 concentrations ranging from 4.5 to 26.9 µg/m^3^, compared to a reference level of 2.0 µg/m^3^. However, most studies included in this review examined exposure levels exceeding current air quality thresholds. Notably, 90% reported mean or median PM2.5 levels above the WHO's annual guideline of 5 µg/m^3^.^15^ Even when using a less stringent benchmark, such as the EU's proposed annual limit of 10 µg/m^3^ by 2030,^16^ over 70% still reported higher exposure levels. As a result, evidence characterizing the exposure–response relationship at lower PM2.5 concentrations, particularly in populations with sustained exposures below major international guideline values, remains comparatively limited.

Studies conducted in regions with lower air pollution levels yielded inconsistent findings. For example, a study of 11,243 males in Australia,^17^ where the mean PM2.5 concentration was 4.54 µg/m^3^, found no significant association between PM2.5 exposure and the incident dementia over a 22.7‐year period. In contrast, a Swedish study^18^ that included 1846 males and females over 21 years of follow‐up, with a mean annual PM2.5 concentration of 6.77 µg/m^3^, reported a 23% increase in dementia risk per 1 µg/m^3^ increase in exposure. These contrasting results highlight ongoing uncertainty regarding the association between low‐level PM2.5 exposure and dementia risk.

Further, sex differences have been documented in brain structure and function, as well as in dementia risk, with Alzheimer's disease (AD) and other dementias occurring more frequently in females than in males.^19^, ^20^ Accumulating evidence also indicates sex‐related differences in key biological processes relevant to neurodegeneration, including neuroinflammation, oxidative stress, cerebrovascular function, and hormonal changes across the life course.^21^, ^22^ Genetic susceptibility also appears to differ by sex; female carriers of the *APOE* ε4 allele have been shown to experience higher risk of AD than male carriers.^23^ Together, these observations raise the question of whether the association between PM2.5 exposure and dementia risk differs by sex.

To address these gaps, we investigated the association between PM2.5 exposure and incident dementia in a large sample of males and females across four states and one territory in Australia, where annual mean PM2.5 levels are relatively low (below the Australian National Environment Protection Measure standard of 8 µg/m^3^).^24^ We examined both continuous exposure levels and categorical cut‐offs based on current air quality guidelines, with the goal of providing potential threshold evidence to inform long‐term dementia prevention strategies. We also assessed whether these associations differed by sex.

## 2 METHODS

### 2.1 ASPREE cohort

**RESEARCH IN CONTEXT**

1. **Systematic review**: The authors reviewed the literature using traditional sources (e.g., PubMed) and conference abstracts. Most previous studies were conducted in more polluted regions, while few examined the association between long‐term PM2.5 exposure and dementia risk in low‐exposure settings. These relevant studies are appropriately cited.
2. **Interpretation**: In an environment with annual mean PM2.5 < 8 µg/m^3^, our findings suggest a sex‐specific association: Females showed increased dementia risk per 1 µg/m^3^ increase in exposure, whereas males did not. The largely null associations in the full cohort align with some prior studies in low‐exposure settings. However, a potential threshold effect emerged at exposure levels >10 µg/m^3^, with stronger vulnerability in females.
3. **Future directions**: These findings highlight the need for replication in other low‐exposure populations, mechanistic studies on sex‐specific vulnerability, and further evaluation of threshold effects to inform air quality standards and dementia prevention strategies.

Data from the Aspirin in Reducing Events in the Elderly (ASPREE) clinical trial and its observational extension study, ASPREE‐XT (with data cut‐off up to the sixth annual visit, 2023 to 2024), were used for this analysis. Full details of the study design and eligibility criteria have been published elsewhere.^25^, ^26^, ^27^, ^28^ In brief, ASPREE was a double‐blind, randomized, placebo‐controlled trial investigating the effects of low‐dose aspirin compared to a matching placebo (registered at Clinicaltrials.gov: NCT01038583). Recruitment took place between March 2010 and December 2014, enrolling 19,114 community‐dwelling participants (16,703 Australian and 2411 US adults) aged over 70 years (or ≥65 for African American and Hispanic participants in the United States). Participants in Australia were recruited through collaborations with over 2000 general practitioners (GPs) and via clinic‐based mailing lists, electronic medical screening, and media advertisements in the United States.^29^ At enrollment, participants were free from cardiovascular disease, dementia, and physical disabilities that limited independent living and had an expected survival of at least 5 years as judged by their treating physician. All participants also had a Modified Mini‐Mental State (3MS) Examination score above 78/100. At the end of the trial in 2017, surviving participants were invited to continue in the observational follow‐up study, ASPREE‐XT, with 93% consenting to do so (16,317 out of 17,546).^30^

For the present analysis, only Australian participants were included, as residential address data required for geocoding, and thus PM2.5 exposure estimation, were unavailable for US participants. During both the ASPREE and ASPREE‐XT studies, comprehensive health data were gathered through annual visits, covering physical function, lifestyle factors, anthropometrics, cognition, disability, hospitalizations, prescription medications, and other related health information. These visits were further supported by interim phone calls – every 3 months during the ASPREE trial phase and every 6 months during the ASPREE‐XT follow‐up phase – to proactively identify potential dementia events.

### 2.2 PM2.5 exposure assessment

Residential addresses of Australian participants were collected at recruitment. These addresses were geocoded using the Geoscape Geocoded National Address File (G‐NAF, May 2024 release) to obtain precise longitude and latitude coordinates. Each record was matched to the G‐NAF “default” geocode, typically the property centroid. Records that showed mismatches (e.g., suburb or postcode inconsistencies, or cases where the address was listed as a building name rather than an actual street) were manually checked. To ensure spatial accuracy, participants whose addresses showed substantial discrepancies (e.g., a mismatch of more than 10 km between the postcode and street location) were excluded from the study. Non‐street addresses (e.g., central PO Boxes in Australia, which could be quite far from an individual's home address) were also excluded. All coordinates were transformed to a common geographic reference system (GDA2020) for consistency.

To ensure appropriate temporal ordering and consistent with prior research on air pollution and dementia,^17^, ^31^, ^32^ cumulative exposure windows were applied. One‐year mean PM2.5 exposure prior to baseline was estimated as the primary exposure of interest, with 3‐year mean exposure included in sensitivity analyses. Daily ambient PM2.5 concentrations from March 2007 to December 2013 (covering the 1 to 3 years before the recruitment period) were obtained from a high‐resolution global dataset (0.1° × 0.1° grid) developed using a deep ensemble machine learning (DEML) approach. Briefly, Yu et al.^33^ estimated global daily PM2.5 concentrations at a 0.1° × 0.1° spatial resolution by training a DEML model from 5446 monitoring stations worldwide, including 167 stations in Australia, and incorporated a wide range of predictors, including meteorological variables (temperature, wind speed, humidity, pressure, and precipitation) and geographical features (land cover, elevation, and population density). Further methodological details regarding the development of the dataset are available in Yu et al.^33^

For each Australian participant, daily PM2.5 exposure was estimated based on their geocoded residential location using one of three spatial interpolation methods, chosen according to the participant's proximity to the grid and data availability. For instance, if a participant's address fell within a valid grid cell, the nearest‐neighbor method was used, assigning the PM2.5 value from that cell. If the address did not fall directly within a grid cell with valid data, bilinear interpolation was applied to compute a weighted average of the four surrounding cells. In cases where bilinear interpolation was not feasible – due to edge effects (e.g., proximity to grid boundaries) or missing data – the PM2.5 value was calculated as the mean of all grid cells within a 2.5‐km radius of the participant's location.

One‐year and 3‐year mean PM2.5 exposure prior to baseline were then calculated for each participant by taking the arithmetic mean of their daily exposure estimates. All data processing was conducted using R statistical software (version 4.4.3).

### 2.3 Incident dementia

Trained and accredited staff conducted cognitive assessments annually throughout the ASPREE and ASPREE‐XT studies. Dementia “triggers,” which prompted further clinical evaluation, included any of the following: a 3MS score below 78,^34^ a decline of more than 10.15 points from the age‐ and education‐adjusted 5‐year predicted 3MS score,^35^ participant‐ or clinician‐reported concerns about memory or cognition, or documentation of a dementia diagnosis or prescription of a cholinesterase inhibitor.

Participants exhibiting any of these triggers underwent additional evaluations. These included, but were not limited to, the Alzheimer's Disease Assessment Scale‐Cognitive subscale (ADAS‐Cog) ^36^ to assess aphasia and apraxia, the Color Trails Test^37^ to assess executive function, and the Alzheimer's Disease Cooperative Study‐Instrumental Activities of Daily Living (ADCS‐IADL) scale,^38^ which was completed by the participant and, if available, a study partner, to assess functional decline.

To minimize the influence of transient conditions such as delirium, follow‐up assessments were administered at least 6 weeks after the initial trigger. When available, additional clinical data relevant to the dementia assessment, such as brain imaging, laboratory or blood test results, and clinician notes regarding suspected cognitive change, were also collected.

A blinded adjudication committee, comprising specialists in neurology, neuropsychology, and geriatrics from Australia and the United States reviewed all available information to determine dementia diagnoses based on Diagnostic and Statistical Manual of Mental Disorders, 4th Edition (DSM‐IV) criteria.^39^ The date of dementia diagnosis was defined as the date of the trigger that ultimately led to a confirmed diagnosis.

### 2.4 Statistical analysis

Statistical analyses for this study were conducted between February 24 and August 28, 2025. The primary exposure of interest was the 1‐year mean PM2.5 exposure prior to baseline. Cox proportional hazards regression models were used to estimate cause‐specific hazard ratios (HRs) and 95% confidence intervals (CIs) for the association between PM2.5 exposure and incident dementia. The proportional hazards assumption was assessed using the Schoenfeld residuals method. Participants were followed from baseline until the earliest occurrence of dementia diagnosis, death, or end of follow‐up.

Initially, a linear exposure–response relationship was assumed, with PM2.5 exposure included as a continuous variable in the Cox models. To explore the potential for non‐linear associations, as suggested by recent literature,^4^ a restricted cubic splines (RCS) model with three knots (positioned at 10th, 50th, and 90th percentiles of the exposure distribution, yielding 2 degrees of freedom) was implemented. Evidence for a non‐linear association of this form was formally evaluated by comparing the RCS model to the linear model using a likelihood ratio test.

Additionally, to explore potential threshold effects, 1‐year mean PM2.5 exposure was categorized based on guideline‐relevant cut‐offs: 5 µg/m^3^ (WHO air quality guideline),^6^ 8 µg/m^3^ (Australian national standard),^40^ and 10 µg/m^3^ (EU recommendation for 2030).^16^ This resulted in four exposure groups: ≤5, >5 to 8, >8 to 10, and >10 µg/m^3^. Cox proportional hazard regression models were re‐estimated using these categorical exposure variables, with ≤5 µg/m^3^ serving as the reference group.

Covariates were selected a priori based on prior literature,^5^, ^41^, ^42^, ^43^ and analyses were conducted using a series of four progressively adjusted models, including (1) an unadjusted model; (2) Model 1, adjusted for basic demographics (age, sex, and education level); (3) Model 2, additionally adjusted for smoking status, alcohol consumption, body mass index (BMI), state of residence, rurality, and socioeconomic status as indicated by Index of Relative Socio‐economic Advantage and Disadvantage (IRSAD) to account for a wider range of known lifestyle and environmental confounders; and (4) Model 3, further adjusted for *APOE* ε4 carrier status to account for genetic susceptibility, in the subsample of participants who agreed to genotyping (*n* = 13,021).

Potential effect modification was assessed by including interaction terms between PM2.5 exposure and several factors known to influence dementia risk or susceptibility ^44^, ^45^: sex (males vs females), *APOE* ε4 status (carriers vs non‐carriers), and age groups (70 to 74, 75 to 80, >80 years); and subgroup analyses stratified by these factors to examine potential heterogeneity in the associations.

All statistical tests were two‐sided, and a *p* value below 0.05 was considered statistically significant. Analyses were conducted using Stata version 17.

### 2.5 Sensitivity analysis

To assess the robustness of the main findings, we conducted three sensitivity analyses: (1) to address the possibility that participants might have died before surviving long enough to be diagnosed with dementia, Fine and Gray models were used to estimate subdistribution HRs for dementia, treating all‐cause death as a competing event; (2) the 1‐year mean PM2.5 exposure was replaced with a 3‐year mean exposure; while longer windows may introduce misclassification if participants move before baseline, a 3‐year mean better reflects cumulative exposure, which is relevant given dementia's long latency; and (3) the follow‐up period was restricted to a shorter duration (approximately the median duration of 5 years) to reduce the window over which unmeasured exposure changes could occur. All sensitivity analysis models were adjusted using the same set of covariates as in the primary analysis.

## 3 RESULTS

### 3.1 Baseline characteristics of participants

Of the 16,235 Australian participants who provided residential addresses at baseline, three were excluded due to vague address information, leaving 16,232 with valid geocodes. An additional 87 participants were excluded because PM2.5 data were unavailable for their geocoded areas. The final analytic sample therefore included 16,145 Australian ASPREE participants, representing 84.5% of the total ASPREE cohort and 96.7% of all Australian participants. Over half (54.87%) were females. At recruitment, participants were aged between 70 and 96 years, with a median age of 74 and a mean age of 75. All participants were without dementia at baseline and with good global cognition, having a mean 3MS score of 93.4 (standard deviation [SD] 4.6). Over half of participants had less than 12 years of completed education, 3.4% were current smokers, and 79.1% reported current alcohol consumption. More than half (52.6%) of participants lived in major Australian cities. The follow‐up period for this study had a median duration of 10.3 years (interquartile range [IQR]: 9.6 to 13.2 years). During follow‐up, 4045 participants (25.1%) died, and 1439 (8.9%), including 692 males and 747 females, were diagnosed with dementia. There were no significant differences in baseline demographic, lifestyle, or spatial distribution factors between participants included versus excluded from the main analysis due to missing PM2.5 exposure data.

Table 1 presents the baseline characteristics of the study population by PM2.5 exposure categories defined by WHO (5 µg/m^3^), Australia (8 µg/m^3^), and EU (10 µg/m^3^) air quality recommendations. Participants exposed to higher PM2.5 concentrations did not differ significantly in education level, BMI, smoking status, alcohol consumption, socioeconomic status, *APOE* genotype, 3MS cognitive scores, or baseline chronic conditions (including hypertension, diabetes, and dyslipidemia). However, they were more likely to be male, younger, and less likely to reside in inner regional areas of Australia. We also examined participants who consented to genetic testing (13, 021; 80% of the full sample). Compared with the full analytic sample, this subsample was more likely to be male, younger, have higher education, drink alcohol, be overweight, and live in major cities but did not differ in smoking status. Within the genotyped subsample, 25.6% were *APOE ε4* carriers (either homozygous or heterozygous), and baseline characteristics by PM2.5 levels were broadly consistent with those in the full sample (Table S1).

**TABLE 1:** Baseline characteristics of participants by levels of annual mean PM2.5.

	≤5 µg/m^3^	>5 to 8 µg/m^3^	>8 to 10 µg/m^3^	>10 µg/m^3^	Overall
***N* (%)**	1,124 (6.96)	10,273 (63.63)	4340 (26.88)	408 (2.53)	16,145 (100)
**Female, *n* (%)**	609 (54.18)	5726 (55.74)	2309 (53.20)	214 (52.45)	8858 (54.87)
**Age, years, *n* (%)**					
70 to 74	608 (54.09)	5832 (56.77)	2610 (60.14)	271 (66.42)	9321 (57.73)
75 to 80	331 (29.45)	2756 (26.83)	1103 (25.41)	85 (20.83)	4275 (26.48)
80+	185 (16.46)	1685 (16.40)	627 (14.45)	52 (12.75)	2549 (15.79)
**Education, years, *n* (%)**					
<12	569 (50.62)	5236 (50.97)	2125 (48.97)	200 (49.02)	8130 (50.36)
12 to 15	299 (26.60)	2663 (25.92)	1144 (26.37)	115 (28.19)	4221 (26.15)
16+	256 (22.78)	2374 (23.11)	1070 (24.66)	93 (22.79)	3793 (23.49)
**BMI, *n* (%)**					
Underweight, <20	28 (2.50)	194 (1.90)	78 (1.81)	2 (0.49)	302 (1.88)
Normal, 20 to 24.9	282 (25.22)	2515 (24.57)	1043 (24.19)	89 (21.98)	3929 (24.45)
Overweight, 25 to 29.9	515 (46.06)	4580 (44.74)	1967 (45.63)	187 (46.17)	7249 (45.11)
Obese, 30+	293 (26.21)	2948 (28.80)	1223 (28.37)	127 (31.36)	4591 (28.57)
**Smoking status, *n* (%)**					
Never	624 (55.52)	5744 (55.91)	2387 (55.00)	212 (51.96)	8967 (55.54)
Former	453 (40.30)	4171 (40.60)	1825 (42.05)	183 (44.85)	6632 (41.08)
Current	47 (4.18)	358 (3.48)	128 (2.95)	13 (3.19)	546 (3.38)
**Alcohol consumption, *n* (%)**					
Never	176 (15.66)	1659 (16.15)	701 (16.15)	59 (14.46)	2595 (16.07)
Former	53 (4.72)	516 (5.02)	197 (4.54)	21 (5.15)	787 (4.87)
Current – low or medium^a^	785 (69.84)	7146 (69.56)	3048 (70.23)	284 (69.61)	11,263 (69.76)
Current – high amount^b^	110 (9.79)	952 (9.27)	394 (9.08)	44 (10.78)	1500 (9.29)
**Rurality, *n* (%)**					
Inner region	419 (37.31)	3790 (37.04)	1422 (32.83)	134 (32.84)	5765 (35.82)
Major cities	571 (50.85)	5273 (51.53)	2420 (55.88)	208 (50.98)	8472 (52.64)
Outer region	133 (11.84)	1170 (11.43)	489 (11.29)	66 (16.18)	1858 (11.54)
**State of residence, *n* (%)**					
VIC	755 (67.17)	7080 (68.92)	2771 (63.85)	227 (55.64)	10,833 (67.10)
NSW/ACT^c^	129 (11.48)	1035 (10.07)	626 (14.42)	77 (18.87)	1867 (11.56)
SA	45 (4.00)	760 (7.40)	553 (12.74)	65 (15.93)	1423 (8.81)
TAS	195 (17.35)	1398 (13.61)	390 (8.99)	39 (9.56)	2022 (12.52)
**IRSAD Quintile** ^d^ **, *n* (%)**					
1 (most disadvantaged)	180 (16.03)	1707 (16.68)	694 (16.02)	77 (18.87)	2658 (16.51)
2	189 (16.83)	1713 (16.74)	771 (17.80)	70 (17.16)	2743 (17.04)
3	201 (17.90)	1984 (19.38)	779 (17.99)	85 (20.83)	3049 (18.94)
4	225 (20.04)	1922 (18.78)	811 (18.73)	67 (16.42)	3025 (18.79)
5 (most advantaged)	328 (29.21)	2910 (28.43)	1276 (29.46)	109 (26.72)	4623 (28.72)
**APOE 𝛆4 status, *n* (%)**					
No APOE ε4	642 (57.12)	6117 (59.54)	2691 (62.00)	240 (58.82)	9690 (60.02)
APOE ε4+	242 (21.53)	2104 (20.48)	892 (20.55)	93 (22.79)	3331 (20.63)
Not Sequenced	240 (21.35)	2052 (19.97)	757 (17.44)	75 (18.38)	3124 (19.35)
**3MS, mean (SD)**	93.48 (4.57)	93.40 (4.58)	93.37 (4.55)	93.17 (4.33)	93.39 (4.56)
**Diabetes, *n* (%)**	103 (9.16)	1011 (9.84)	435 (10.02)	43 (10.54)	1592 (9.86)
**Dyslipidemia, *n* (%)**	777 (69.13)	6987 (68.01)	2879 (66.34)	278 (68.14)	10,921 (67.64)
**Hypertension, *n* (%)**	847 (75.36)	7745 (75.39)	3230 (74.42)	292 (71.57)	12,114 (75.03)

Abbreviations: 3MS, Modified Mini‐Mental State Examination; ACT, Australian Capital Territory; APOE ε4, apolipoprotein E with ε4 allele; BMI, body mass index; IRSAD, Index of Relative Socio‐economic Advantage and Disadvantage; NSW, New South Wales; SA, South Australia; SD, standard deviation; TAS, Tasmania; VIC, Victoria.

^a^ Low or medium alcohol consumption was defined as drinking ≤once per week and consuming one to 12 standard drinks per occasion, 1 to 2 days per week and consuming one to eight standard drinks per occasion, 3 to 4 days per week and consuming one to four standard drinks per occasion, or ≥5 days per week and consuming one to two standard drinks per occasion.

^b^ High alcohol consumption was defined as drinking 1 to 2 days per week and consuming nine to 12 standard drinks per occasion, 3 to 4 days per week and consumes five to 12 standard drinks per occasion, ≥5 days per week and consuming three to 12 standard drinks per occasion, or consuming ≥13 drinks, regardless of frequency.

^c^ Participants living in ACT and NSW are combined into a single group for two reasons: The ACT is geographically located within NSW and therefore shares similar environmental conditions with nearby inland NSW regions, such as temperature and humidity; additionally, the small sample size in the ACT limits statistical power.

^d^ The IRSAD is one of the Socio‐Economic Indexes for Areas developed by the Australian Bureau of Statistics. It is based on census data such as income, education, employment, occupation, and housing characteristics, and summarizes the relative socio‐economic advantage and disadvantage of an area.

### 3.2 PM2.5 exposure

**FIGURE 1:**
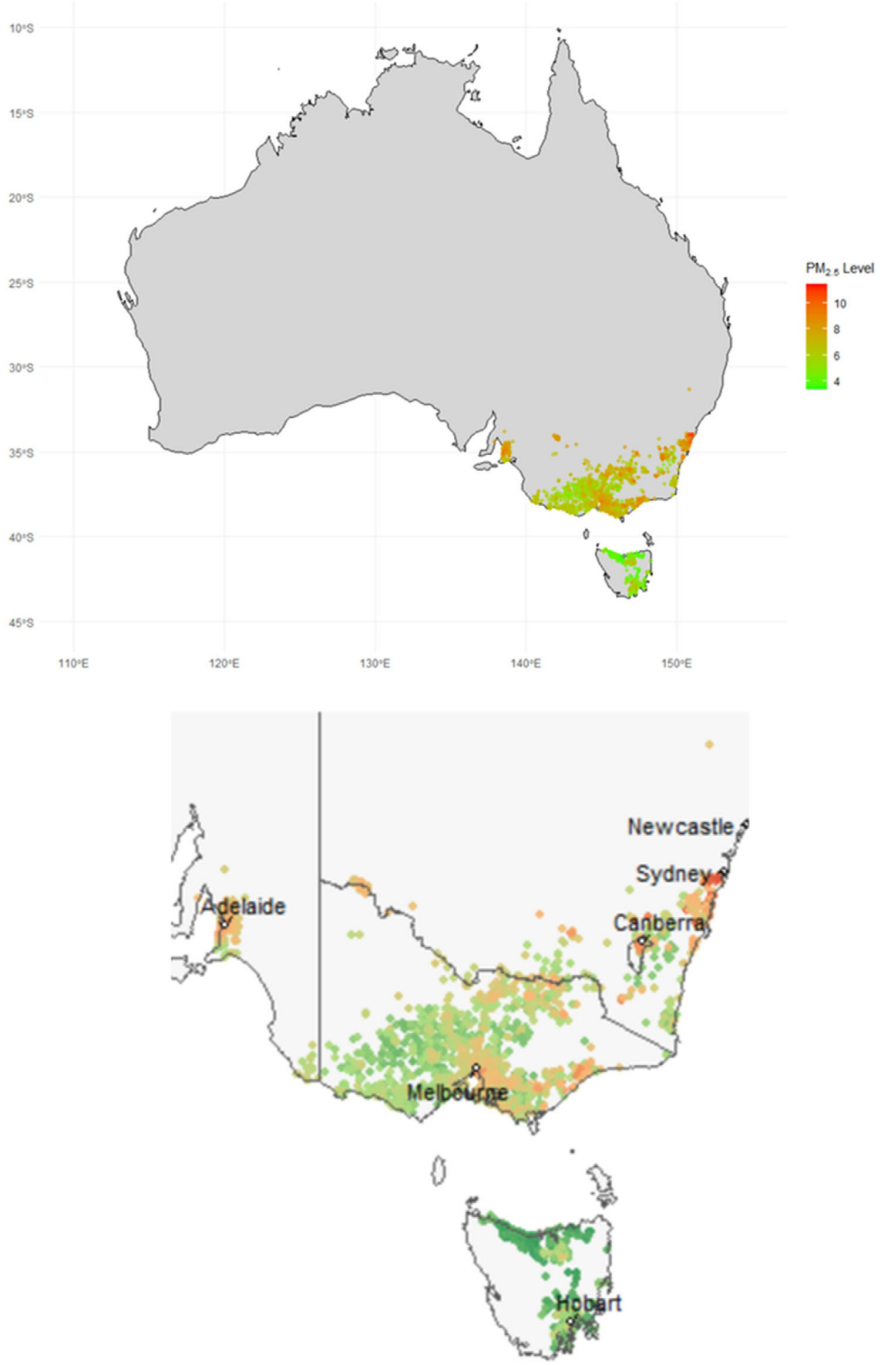
One‐year mean PM2.5 exposure before baseline by residential address in Aspirin in Reducing Events in the Elderly (ASPREE) participants.

Figure 1 shows the spatial distribution of estimated 1‐year mean PM2.5 concentrations at participants’ residential addresses prior to baseline. Participants were located across four Australian states – Victoria (VIC, 67.0%), Tasmania (TAS, 12.5%), South Australia (SA, 8.8%), and New South Wales (NSW, 7.5%) – and the Australian Capital Territory (ACT, 4.2%). One‐year mean PM2.5 exposure was 7.3 µg/m^3^ (SD: 1.4; median: 7.3, IQR: 6.6 to 8.2), ranging from 3.4 µg/m^3^ to 11.4 µg/m^3^.

### 3.3 Association of PM2.5 with risk of dementia

In Cox proportional hazards regression models, no statistically significant associations were observed between PM2.5 exposure, assessed both as a continuous variable and using guideline‐based cut‐offs, and incident dementia in the overall population (Table 2). In the adjusted model (Model 2), the estimated hazard ratio per 1 µg/m^3^ increase in PM2.5 was 1.01 (95% CI: 0.97 to 1.05, *p* = 0.58) and 1.04 (95% CI: 0.99 to 1.08, *p* = 0.11) in the fully adjusted model, including *APOE Ɛ4* genotype (Model 3). Schoenfeld residuals indicated no evidence of violation of the proportional hazard assumption for PM2.5 exposure across all models. The likelihood ratio test provided no evidence of non‐linearity. When exposure was categorized, the highest risk was seen in the >10 µg/m^3^ group compared with ≤5 µg/m^3^, corresponding to a 50% increased risk of dementia in the fully adjusted model; however, this was not statistically significant (HR 1.50, 95% CI: 0.97 to 2.31, *p* = 0.07).

**TABLE 2:** Cox proportional hazards models for the association between PM2.5 exposure and incident dementia in the overall population.

Models	PM2.5 exposure (continuous, µg/m^3^)		PM2.5 exposure (cut‐offs, µg/m^3^)		
(*N* of obs)	HR (95% CI)	*P* value		HR (95% CI)	*P* value
Unadjusted	1.01 (0.97, 1.04)	0.80	≤5	1.00 (Reference)	
(16,145)			>5 to 8	1.04 (0.84, 1.27)	0.74
			>8 to 10	1.01 (0.81, 1.25)	0.96
			>10	1.23 (0.84, 1.80)	0.28
Model 1^a^	1.02 (0.98, 1.05)	0.42	≤5	1.00 (Reference)	
(16,144)			>5 to 8	1.05 (0.85, 1.28)	0.67
			>8 to 10	1.05 (0.84, 1.31)	0.65
			>10	1.34 (0.91, 1.96)	0.13
Model 2^b^	1.01 (0.97, 1.05)	0.58	≤5	1.00 (Reference)	
(16,021)			>5 to 8	1.05 (0.85, 1.29)	0.65
			>8 to 10	1.04 (0.83, 1.30)	0.74
			>10	1.34 (0.91, 1.96)	0.14
Model 3^c^	1.04 (0.99, 1.08)	0.11	≤5	1.00 (Reference)	
(12,930)			>5 to 8	1.11 (0.87, 1.41)	0.40
			>8 to 10	1.15 (0.89, 1.48)	0.30
			>10	1.50 (0.97, 2.31)	0.07

Abbreviations: CI, confidence interval; HR, hazard ratio

^a^ Adjusted for age, sex, and education level.

^b^ Adjusted for age, sex, education level, smoking status, alcohol consumption, body mass index (BMI), state of residence, rurality, and socioeconomic status as indicated by Index of Relative Socio‐economic Advantage and Disadvantage.

^c^ Adjusted for age, sex, and education level, smoking status, alcohol consumption, body mass index (BMI), state of residence, rurality, and socioeconomic status as indicated by Index of Relative Socio‐economic Advantage and Disadvantage (IRSAD), and APOE ε4 genotype.

### 3.4 Sex‐specific associations between PM2.5 exposure and incident dementia

**FIGURE 2:**
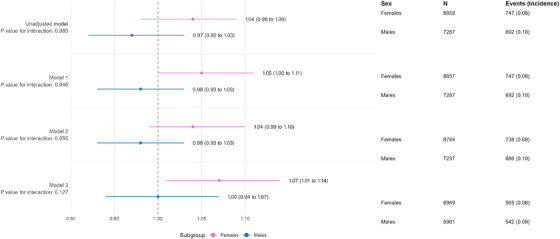
Cox proportional hazards models for association between PM2.5 exposure (per unit increase) and incident dementia: subgroup analysis by sex.

**FIGURE 3:**
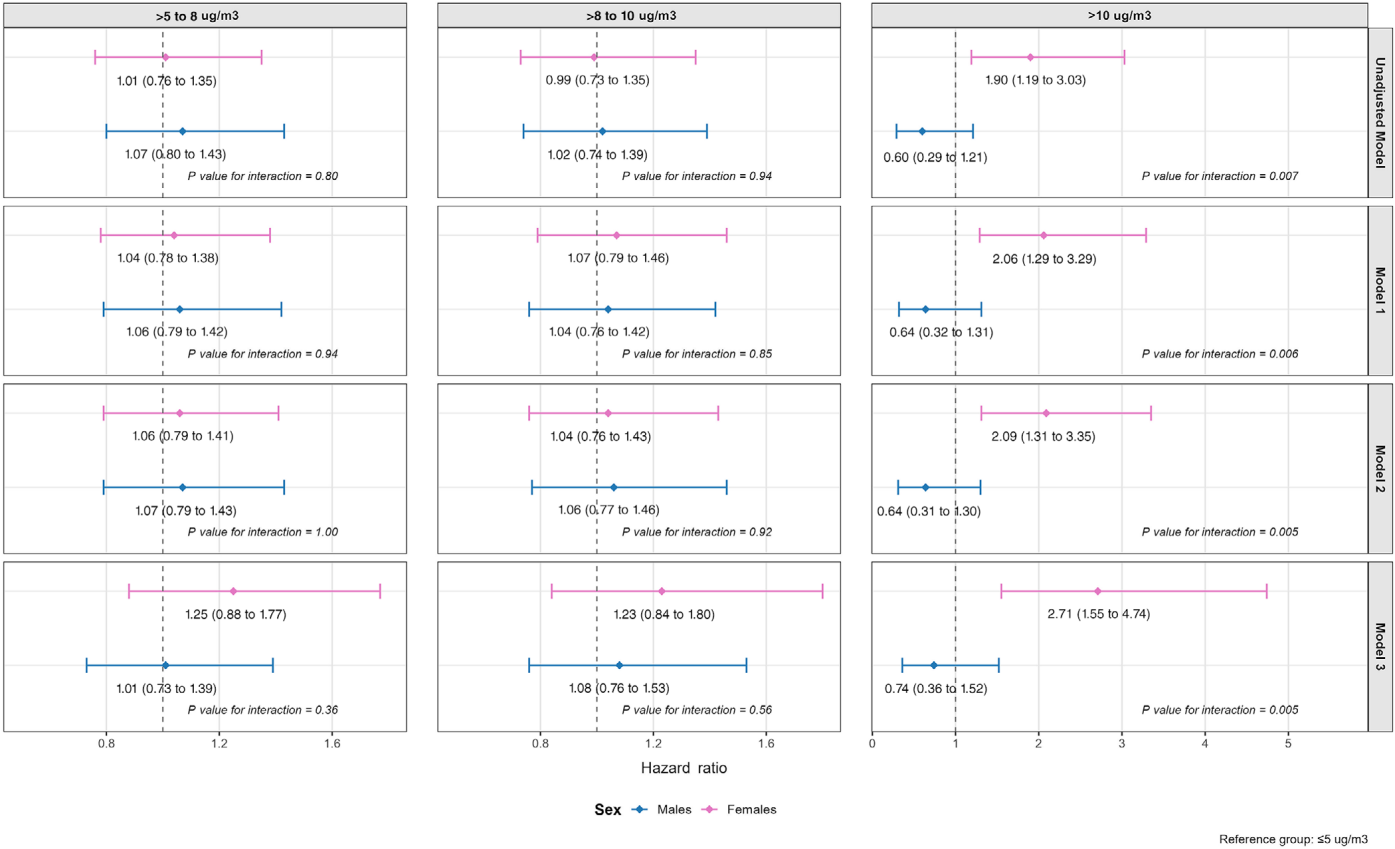
Cox proportional hazards models for association between PM2.5 exposure (cut‐offs) and incident dementia: subgroup analysis by sex.

There was some evidence that sex modified the association between air pollution and dementia, with interaction terms for continuous exposures ranging from 0.046 to 0.13 across all models (Figure 2). When exposure was categorized, the interaction with sex in the highest exposure group was statistically significant across all models (*p* values ranging from 0.005 to 0.007; Figure 3), whereas no evidence of interaction was observed in the lower categories of exposure.

Analyses stratified by sex revealed different patterns of associations. In females (Table S2), the association estimates were consistently positive across all models, reaching statistical significance in the fully adjusted model (Model 3), where each 1 µg/m^3^ increase in 1‐year mean PM2.5 exposure was associated with a 7% higher risk of dementia (HR 1.07, 95% CI: 1.01 to 1.14, *p* = 0.03).

Similar trends were observed when PM2.5 exposure was categorized using air quality guideline cut‐offs. In females, exposure levels between 5 and 8 µg/m^3^ and 8 and 10 µg/m^3^ were associated with modest, non‐significant increases in dementia risk compared to the reference group of ≤5 µg/m^3^ (HRs around 1.2 in the fully adjusted model). However, exposure above 10 µg/m^3^ was associated with a markedly higher risk: Females in this group had approximately a 2.7‐fold increased risk of dementia (Model 3: HR 2.71, 95% CI: 1.55 to 4.74, *p* < 0.001). In contrast, no significant associations were observed in males for either continuous or categorical PM2.5 exposure, and point estimates remained close to null (Table S3).

Subgroup analyses by *APOE ε4* status (*APOE ε4* carriers vs non‐carriers; Table S4) and age group (70 to 74, 75 to 80, ≥80 years; Table S5) did not reveal any significant differences in the association between PM2.5 exposure and dementia.

In sensitivity analyses accounting for death as a competing risk, the effect sizes were smaller but remained directionally consistent (Tables S6–S8). Among females in the highest exposure group (> 10 µg/m^3^), there was a 2.5‐fold increased risk of dementia (Model 3: Subdistribution Hazard Ratio (SHR) 2.47, 95% CI: 1.41 to 4.32, *p* = 0.003), with a similar trend observed for each unit increase in PM2.5 exposure (Model 3: SHR 1.06, 95% CI: 0.99 to 1.12, *p* = 0.08; Table S7). Similarly, no associations were observed in males (Table S8). In sensitivity analyses using 3‐year mean PM2.5 exposures prior to baseline instead of the 1‐year mean (Tables S9–S11), the findings were in the same direction but attenuated and no longer statistically significant. Sensitivity analyses restricting the follow‐up to a shorter duration yielded results consistent with the primary analyses (Tables S12–S14), with slightly stronger effect estimates in the overall population (Model 3: HR 1.08, 95% CI: 0.99 to 1.17, *p* = 0.072; Table S12) and among females (Model 3: SHR 1.13, 95% CI: 1.01 to 1.27, *p* = 0.037; Table S13), while associations remained null in males (Table S14). When PM2.5 exposure was categorized, 95% CIs for males in the highest exposure group were not estimable, likely due to the smaller number of dementia cases identified during the shorter follow‐up period.

## 4 DISCUSSION

In this large cohort of healthy, community‐dwelling older adults living in southeastern Australia, a region with relatively low air pollution, we observed no significant association between long‐term PM2.5 exposure and 14‐year incident dementia in the overall population. However, analyses suggested a possible threshold effect: Individuals exposed to PM2.5 levels above 10 µg/m^3^ had a 50% higher risk of dementia after adjusting for a broad range of demographic, lifestyle, environmental, and genetic factors. In sex‐stratified analyses, this association was more pronounced in females. Specifically, exposure above 10 µg/m^3^ was associated with a 2.7‐fold increase in risk compared to levels below 5 µg/m^3^. Consistent with this pattern, each 1 µg/m^3^ increase in PM2.5 exposure was associated with a 7% higher dementia risk in females but not males. This large study, with rigorous ascertainment of incident dementia and high‐resolution exposure estimates, contributes important evidence and highlights a possible threshold effect for exposure levels and sex‐specific vulnerability.

Our null findings in the overall population can be compared to a limited number of prospective studies conducted in similarly low‐pollution settings, where mean or median PM2.5 levels were under or close to the current study exposure levels of 7.5 µg/m^3^.^17^, ^31^, ^32^, ^46^, ^47^ For instance, Yuchi et al. ^46^ used administrative health data from 678,000 individuals aged 45 to 84 in Vancouver Canada, where medium PM2.5 was approximately 7.5 µg/m^3^. They reported no association between PM2.5 and risk of either Alzheimer's dementia (OR 0.90, 95% CI: 0.76 to 1.07 per 1.5 µg/m^3^ IQR increase) or non‐Alzheimer's dementia (HR 1.02, 95% CI: 0.98 to 1.05). In contrast, Smargiassi et al. ^32^ used linked medico‐administrative databases of 1.8 million older adults in Quebec, Canada, with a median PM2.5 of 7.9 µg/m^3^. They found a 2% increased risk of dementia per 3.9 µg/m^3^ increase (HR 1.02, 95% CI: 1.00 to 1.03). Our findings align closely with these studies, with null or weak associations, and suggest that the adverse cognitive effects of PM2.5 may be less evident at lower exposure levels.

On the other hand, most previous studies reporting significant associations between PM2.5 and dementia were conducted in higher‐exposure regions. For example, studies from Taiwan (mean annual PM2.5: 33.6 µg/m^3^),^48^ Hong Kong (35.2 µg/m^3^),^49^ and Montpellier, France (14.6 to 31.3 µg/m^3^)^50^ consistently found increased dementia risk associated with PM2.5 exposure in combined male and female samples. Even in studies conducted in lower‐exposure settings (but still above the exposure level in our study), increased dementia risk was also observed.^51^, ^52^, ^53^ In our cohort, only a small proportion of participants (2.5%) were exposed to PM2.5 levels exceeding 10 µg/m^3^, the limit proposed by the EU as its 2030 target. While associations above this threshold did not reach statistical significance in our overall sample, there was a suggestion of increased dementia risk at these higher levels. Thus, taken together with prior research, our findings support the possibility that only relatively high levels of PM2.5 may significantly impact dementia risk, thereby reinforcing the importance of identifying potential thresholds for dementia prevention in low‐pollution settings.

Very few prior studies investigated possible sex‐differences in the association between air pollution and dementia, but in general, these studies reported stronger associations in females than in males.^32^, ^54^, ^55^ Furthermore, some studies involved single‐sex populations, and their findings align with ours.^17^, ^56^, ^57^ For example, a study of 3647 older females followed for up to 11 years found that living in areas where PM2.5 levels exceeded 12 µg/m^3^ was associated with significantly increased risk of all‐cause dementia (by 92%).^56^ These converging findings suggest that females may be more susceptible to PM2.5‐related neurotoxicity, consistent with the patterns observed in the current study even after adjustment for established risk factors, with the sex difference becoming more apparent at higher exposure levels. Importantly, results from competing risk models were consistent with the primary analyses, suggesting that the observed sex differences are unlikely due to differential mortality by sex.

This heightened susceptibility in females may reflect underlying biological differences.^21^ Females have been shown to exhibit stronger immune and inflammatory responses than males,^58^ which may be relevant in the context of environmental exposures associated with neuroinflammation. Moreover, postmenopausal females may face greater vulnerability due to the loss of estrogen, a hormone with well‐established antioxidant, anti‐inflammatory, and vascular protective effects.^59^, ^60^, ^61^ The decline in estrogen levels during menopause may reduce the brain's resilience to environmental stressors,^62^, ^63^ including PM2.5 exposure. While our study cannot disentangle these biological processes, the observed sex‐specific associations are consistent with prior evidence indicating heterogeneity in dementia risk between females and males. Together, these findings underscore the importance of considering sex as a potential effect modifier in future research on air pollution and dementia.

Across three sensitivity analyses, our findings were directionally consistent with the primary analysis, strengthening confidence in the robustness of our findings. Notably, associations were attenuated when using a 3‐year mean exposure, which may reflect increased exposure misclassification, as longer averaging windows are more likely to be affected by residential mobility that we could not account for. Conversely, recent research found little evidence that earlier exposures were more relevant for incident dementia; instead, stronger associations have been observed for more recent exposures, a pattern consistent with our findings.^64^

A key strength of this study is the use of high‐quality data from the ASPREE and ASPREE‐XT studies. This large‐scale, population‐based cohort provided several advantages: A substantial sample size enabling sex‐stratified and subgroup analyses; a well‐defined population of community‐dwelling older adults who were dementia‐free at baseline and followed for up to 14 years; and comprehensive data on a wide range of dementia‐associated covariates, including *APOE* genotype. Importantly, dementia outcomes were ascertained using an active, rigorous follow‐up process that included annual cognitive assessments, evidentiary clinical documentation, and blind adjudication based on DSM‐IV criteria. This minimizes misclassification and strengthens the reliability of our findings compared with studies relying on linkage, hospital, or other administrative data. Another major strength is the precise and methodologically robust assessment of individual‐level PM2.5 exposure. Residential addresses were geocoded with high spatial accuracy, and participants with major discrepancies were excluded to reduce misclassification. Daily PM2.5 concentrations were obtained from a validated, high‐resolution (0.1° × 0.1°) global dataset developed using a deep ensemble machine learning model. Exposure estimates were then tailored to each participant's location using an adaptive interpolation strategy, ensuring accurate and spatially relevant exposure assignment. Finally, we conducted comprehensive subgroup analyses to explore potential effect modification by demographic and genetic characteristics. Together, these methodological strengths enhance the robustness and interpretability of our findings.

We also acknowledge several potential limitations of this study. First, our interpretation was primarily based on the fully adjusted model that included 80% of the sample with available *APOE ε4* genotype data. This subsample differed slightly in certain characteristics compared with the full analytic sample, which may limit the generalizability of the findings. However, the overall exposure–outcome patterns were consistent with those observed in the larger sample. Second, potential exposure misclassification remains an important consideration. PM2.5 exposure was estimated at the residential addresses and therefore did not capture individual time‐activity patterns, small‐scale spatial variability within grid cells, or indoor PM2.5 exposures. In addition, exposure changes or episodic extreme pollution events were not explicitly modeled during follow‐up. These factors may contribute to non‐differential exposure misclassification. However, this misclassification is likely limited and predominantly non‐differential. Data from the global PM2.5 dataset used in this study^33^ indicate that annual mean PM2.5 concentrations in Australia remained relatively low, with only a modest increase during 2010 to 2019. Moreover, restricting the duration of follow‐up to reduce the window for unmeasured exposure changes yielded consistent findings. Furthermore, the PM2.5 estimation model demonstrated high predictive performance when validated against independent monitoring data (*R* ^2^ = 0.91),^33^ indicating a strong ability to capture regional‐scale spatial variability in ambient PM2.5. Prior evidence also reported a moderate to strong correlation between indoor and outdoor PM2.5 concentrations,^65^ and within‐grid near‐source increments (e.g., proximity to major roadways) tend to be modest when exposures are averaged over longer time windows such as daily or annual means, with regional background PM2.5 dominating long‐term exposure contrasts.^66^, ^67^ Taken together, any potential exposure misclassification is likely to have attenuated effect estimates to a limited extent. Accordingly, the overall null association observed between long‐term PM2.5 exposure and dementia risk in this low‐exposure population should be interpreted as conservative, and a true association cannot be excluded. Future studies incorporating refined individual‐level exposure assessment or enhanced characterization of episodic pollution events are warranted. Third, we did not distinguish between dementia subtypes, and PM2.5 particles may be expected to contribute more to vascular dementia given their role in cerebrovascular dysfunction and disruption of the blood‐brain barrier.^68^, ^69^ However, in older age, mixed dementia pathologies become much more common.

Further research in low‐exposure settings is needed to more precisely define safe thresholds for dementia prevention. In addition, investigation into sex differences in vulnerability may enhance our understanding of dementia pathology and inform targeted public health strategies.

## CONFLICT OF INTEREST STATEMENT

AO reports being a member of the National Health and Medical Research Council of Australia's Nutrient Reference Value Steering Advisory Committee. JR reports being the co‐chair of the Dementia Australia Research Foundation Scientific Panel and a member of the Victoria Coronial Council. None of these are directly related to the work in this manuscript. All other authors report no disclosures. Author disclosures are available in the supporting information.

## CONSENT STATEMENT

All participants provided written informed consent.

## Supporting information



Supporting information

Supporting information
