# Supplementary material for: The sex‐specific association between long‐term PM2.5 exposure and incident dementia in community‐dwelling older adults in Australia
Source: Alzheimers Dement. 2026 Mar 4;22(3):e71256. doi: 10.1002/alz.71256 (PMC12959250; doi:10.1002/alz.71256)
Supplement: Supplementary file 1 — Supporting information [file ALZ-22-e71256-s001.docx]

Supplementary Table 1. Baseline characteristics of participants with genotype data by levels of annual mean PM2.5

|  | **≤5 µg/m^3^** | **>5-8 µg/m^3^** | **>8-10 µg/m^3^** | **>10 µg/m^3^** | **Overall** |
| --- | --- | --- | --- | --- | --- |
| **N (%)** | 884 (6.79) | 8,221 (63.14) | 3,583 (27.52) | 333 (2.56) | 13,021 (100) |
| **Female, n (%)** | 471 (53.28) | 4,504 (54.79) | 1,861 (51.94) | 164 (49.25) | 7,000 (53.76) |
| **Age, y, n (%)** |  | | | | |
| 70-74 | 485 (54.86) | 4,819 (58.62) | 2,204 (61.51) | 229 (68.77) | 7,737 (59.42) |
| 75-80 | 265 (29.98) | 2,180 (26.52) | 907 (25.31) | 64 (19.22) | 3,416 (26.23) |
| 80+ | 134 (15.16) | 1,222 (14.86) | 472 (13.17) | 40 (12.01) | 1,868 (14.35) |
| **Education, y, n (%)** |  | | | |  |
| <12 | 422 (47.74) | 4,096 (49.82) | 1,733 (48.37) | 160 (48.05) | 6,411 (49.24) |
| 12-15 | 249 (28.17) | 2,130 (25.91) | 948 (26.46) | 96 (28.83) | 3,423 (26.29) |
| 16+ | 213 (24.10) | 1,995 (24.27) | 902 (25.17) | 77 (23.12) | 3,187 (24.48) |
| **BMI, n (%)** |  | | | |  |
| Underweight, <20 | 20 (2.28) | 141 (1.72) | 61 (1.71) | 1 (0.30) | 223 (1.72) |
| Normal, 20-24.9 | 228 (25.94) | 1,974 (24.09) | 848 (23.81) | 71 (21.52) | 3,121 (24.07) |
| Overweight, 25-29.9 | 409 (46.53) | 3,726 (45.47) | 1,647 (46.25) | 160 (48.48) | 5,942 (45.83) |
| Obese, 30+ | 222 (25.26) | 2,354 (28.72) | 1,005 (28.22) | 98 (29.70) | 3,679 (28.38) |
| **Smoking Status, n (%)** |  | | | |  |
| Never | 486 (54.98) | 4,602 (55.98) | 1,989 (55.51) | 173 (51.95) | 7,250 (55.68) |
| Former | 361 (40.84) | 3,356 (40.82) | 1,499 (41.84) | 149 (44.74) | 5,365 (41.20) |
| Current | 37 (4.19) | 263 (3.20) | 95 (2.65) | 11 (3.30) | 406 (3.12) |
| **Alcohol Consumption, n (%)** |  | | | |  |
| Never | 115 (13.01) | 1,292 (15.72) | 557 (15.55) | 50 (15.02) | 2,014 (15.47) |
| Former | 36 (4.07) | 401 (4.88) | 155 (4.33) | 19 (5.71) | 611 (4.69) |
| Current - Low or Medium ^a^ | 643 (72.74) | 5,751 (69.95) | 2,545 (71.03) | 228 (68.47) | 9,167 (70.40) |
| Current - High amount ^b^ | 90 (10.18) | 777 (9.45) | 326 (9.10) | 36 (10.81) | 1,229 (9.44) |
| **Rurality, n (%)** |  | | | |  |
| Inner region | 315 (35.67) | 2,995 (36.56) | 1,144 (31.98) | 101 (30.33) | 4,555 (35.08) |
| Major cities | 477 (54.02) | 4,356 (53.17) | 2,017 (56.39) | 174 (52.25) | 7,024 (54.09) |
| Outer region | 91 (10.31) | 841 (10.27) | 416 (11.63) | 58 (17.42) | 1,406 (10.83) |
| **State of Residence, n (%)** |  | | | |  |
| VIC | 619 (70.02) | 5,770 (70.19) | 2,330 (65.03) | 189 (56.76) | 8,908 (68.41) |
| NSW/ACT ^c^ | 101 (11.43) | 825 (10.04) | 486 (13.56) | 62 (18.62) | 1,474 (11.32) |
| SA | 33 (3.73) | 637 (7.75) | 462 (12.89) | 52 (15.62) | 1,184 (9.09) |
| TAS | 131 (14.82) | 989 (12.03) | 305 (8.51) | 30 (9.01) | 1,455 (11.17) |
| **IRSAD Quintile ^d^, n (%)** |  |  |  |  |  |
| 1 (most disadvantaged) | 116 (13.14) | 1,243 (15.17) | 542 (15.15) | 62 (18.62) | 1,963 (15.12) |
| 2 | 144 (16.31) | 1,377 (16.81) | 638 (17.84) | 55 (16.52) | 2,214 (17.05) |
| 3 | 159 (18.01) | 1,613 (19.69) | 640 (17.89) | 72 (21.62) | 2,484 (19.13) |
| 4 | 184 (20.84) | 1,549 (18.91) | 673 (18.81) | 48 (14.41) | 2,454 (18.90) |
| 5 (most advantaged) | 280 (31.71) | 2,410 (29.42) | 1,084 (30.30) | 96 (28.83) | 3,870 (29.80) |
| **3MS, mean (SD)** | 93.66 (4.39) | 93.62 (4.42) | 93.52 (4.48) | 93.25 (4.30) | 93.58 (4.43) |
| **Diabetes, n (%)** | 72 (8.14) | 802 (9.76) | 345 (9.63) | 35 (10.51) | 1,254 (9.63) |
| **Dyslipidemia, n (%)** | 613 (69.34) | 5,574 (67.80) | 2,366 (66.03) | 223 (66.97) | 8,776 (67.40) |
| **Hypertension, n (%)** | 671 (75.90) | 6,128 (74.54) | 2,655 (74.10) | 237 (71.17) | 9,691 (74.43) |

^a^: Low or medium alcohol consumption was defined as: individuals who drink ≤ once per week and consume 1-12 standard drinks per occasion, or 1-2 days per week and consumes 1-8 standard drinks per occasion, or 3-4 days per week and consumes 1-4 standard drinks per occasion, or ≥5 days per week and consumes 1-2 standard drinks per occasion.

^b^: High alcohol consumption was defined as: individuals who drink 1-2 days per week and consumes 9-12 standard drinks per occasion, or 3-4 days per week and consumes 5-12 standard drinks per occasion, or ≥5 days per week and consumes 3-12 standard drinks per occasion, or anyone who consumes ≥13 drinks, regardless of frequency.

^c^: participants living in ACT and NSW are combined into a single group for two reasons: the ACT is geographically located within NSW and therefore shares similar environmental conditions with nearby inland NSW regions, such as temperature and humidity; additionally, the small sample size in the ACT limits statistical power.

^d^: The Index of Relative Socio-economic Advantage and Disadvantage (IRSAD) is one of the Socio-Economic Indexes for Areas (SEIFA) developed by the Australian Bureau of Statistics (ABS). It is based on census data such as income, education, employment, occupation, and housing characteristics, and summarises the relative socio-economic advantage and disadvantage of an area.

Supplementary Table 2. Cox proportional hazards models for the association between PM2.5 exposure and incident dementia in females

| **Models** | **PM2.5 Exposure (Continuous, µg/m^3^)** | | |  | **PM2.5 Exposure (Cut-offs, µg/m^3^)** | | |
| --- | --- | --- | --- | --- | --- | --- | --- |
| **(N of obs)** |  | **HR (95% CI)** | **P-value** |  |  | **HR (95% CI)** | **P-value** |
| Unadjusted |  | 1.04 (0.98, 1.09) | 0.18 |  | ≤5 | 1.00 (Reference) |  |
| (8,856) |  |  |  |  | >5-8 | 1.01 (0.76, 1.35) | 0.93 |
|  |  |  |  |  | >8-10 | 0.99 (0.73, 1.35) | 0.97 |
|  |  |  |  |  | >10 | 1.90 (1.19, 3.03) | **0.007** |
| Model 1 ^a^ |  | 1.05 (1.00, 1.11) | 0.06 |  | ≤5 | 1.00 (Reference) |  |
| (8,855) |  |  |  |  | >5-8 | 1.04 (0.78, 1.38) | 0.80 |
|  |  |  |  |  | >8-10 | 1.07 (0.79, 1.46) | 0.67 |
|  |  |  |  |  | >10 | 2.06 (1.29, 3.29) | **0.002** |
| Model 2 ^b^ |  | 1.04 (0.99, 1.10) | 0.11 |  | ≤5 | 1.00 (Reference) |  |
| (8,784) |  |  |  |  | >5-8 | 1.06 (0.79, 1.41) | 0.71 |
|  |  |  |  |  | >8-10 | 1.04 (0.76, 1.43) | 0.80 |
|  |  |  |  |  | >10 | 2.09 (1.31, 3.35) | **0.002** |
| Model 3 ^c^ |  | 1.07 (1.01, 1.14) | **0.03** |  | ≤5 | 1.00 (Reference) |  |
| (6,949) |  |  |  |  | >5-8 | 1.25 (0.88, 1.77) | 0.22 |
|  |  |  |  |  | >8-10 | 1.23 (0.84, 1.80) | 0.28 |
|  |  |  |  |  | >10 | 2.71 (1.55, 4.74) | **<0.001** |

^a^: Adjusted for age, sex, and education level.

^b^: Adjusted for age, sex, education level, smoking status, alcohol consumption, body mass index (BMI), state of residence, rurality, and socioeconomic status as indicated by Index of Relative Socio-economic Advantage and Disadvantage (IRSAD).

^c^: Adjusted for age, sex, and education level, smoking status, alcohol consumption, body mass index (BMI), state of residence, rurality, and socioeconomic status as indicated by Index of Relative Socio-economic Advantage and Disadvantage (IRSAD), and APOE ε4 genotype.

Supplementary Table 3. Cox proportional hazards models for the association between PM2.5 exposure and incident dementia in males

| **Models** | **PM2.5 Exposure (Continuous, µg/m^3^)** | | |  | **PM2.5 Exposure (Cut-offs, µg/m^3^)** | | |
| --- | --- | --- | --- | --- | --- | --- | --- |
| **(N of obs)** |  | **HR (95% CI)** | **P-value** |  |  | **HR (95% CI)** | **P-value** |
| Unadjusted |  | 0.97 (0.92, 1.03) | 0.31 |  | ≤5 | 1.00 (Reference) |  |
| (7,286) |  |  |  |  | >5-8 | 1.07 (0.80, 1.43) | 0.66 |
|  |  |  |  |  | >8-10 | 1.02 (0.74, 1.39) | 0.93 |
|  |  |  |  |  | >10 | 0.60 (0.29, 1.21) | 0.15 |
| Model 1 ^a^ |  | 0.98 (0.93, 1.03) | 0.42 |  | ≤5 | 1.00 (Reference) |  |
| (7,286) |  |  |  |  | >5-8 | 1.06 (0.79, 1.42) | 0.71 |
|  |  |  |  |  | >8-10 | 1.04 (0.76, 1.42) | 0.83 |
|  |  |  |  |  | >10 | 0.64 (0.32, 1.31) | 0.22 |
| Model 2 ^b^ |  | 0.98 (0.93, 1.03) | 0.42 |  | ≤5 | 1.00 (Reference) |  |
| (7,237) |  |  |  |  | >5-8 | 1.07 (0.79, 1.43) | 0.67 |
|  |  |  |  |  | >8-10 | 1.06 (0.77, 1.46) | 0.74 |
|  |  |  |  |  | >10 | 0.64 (0.31, 1.30) | 0.21 |
| Model 3 ^c^ |  | 1.00 (0.95, 1.07) | 0.91 |  | ≤5 | 1.00 (Reference) |  |
| (5,981) |  |  |  |  | >5-8 | 1.01 (0.73, 1.39) | 0.97 |
|  |  |  |  |  | >8-10 | 1.08 (0.76, 1.53) | 0.66 |
|  |  |  |  |  | >10 | 0.74 (0.36, 1.52) | 0.41 |

^a^: Adjusted for age, sex, and education level.

^b^: Adjusted for age, sex, education level, smoking status, alcohol consumption, body mass index (BMI), state of residence, rurality, and socioeconomic status as indicated by Index of Relative Socio-economic Advantage and Disadvantage (IRSAD).

^c^: Adjusted for age, sex, and education level, smoking status, alcohol consumption, body mass index (BMI), state of residence, rurality, and socioeconomic status as indicated by Index of Relative Socio-economic Advantage and Disadvantage (IRSAD), and APOE ε4 genotype.

Supplementary Table 4. Cox proportional hazards models for the association between PM2.5 exposure and incident dementia by APOE ε4 genotypes

| **Models**  **(N of obs)** | **PM2.5 Exposure (Continuous, µg/m^3^)** | | |  | **PM2.5 Exposure (Cut-offs, µg/m^3^)** | | |
| --- | --- | --- | --- | --- | --- | --- | --- |
|  |  | **HR (95% CI)** | **P-value** |  |  | **HR (95% CI)** | **P-value** |
| **APOE ε4 non-carriers** | |  |  |  |  |  |  |
| Unadjusted |  | 1.02 (0.96, 1.08) | 0.49 |  | ≤5 | 1.00 (Reference) |  |
| (9,690) |  |  |  |  | >5-8 | 1.14 (0.82, 1.58) | 0.44 |
|  |  |  |  |  | >8-10 | 1.14 (0.81, 1.62) | 0.45 |
|  |  |  |  |  | >10 | 1.25 (0.68, 2.31) | 0.47 |
| Model 1 ^a^ |  | 1.03 (0.98, 1.09) | 0.25 |  | ≤5 | 1.00 (Reference) |  |
| (9,690) |  |  |  |  | >5-8 | 1.16 (0.83, 1.61) | 0.38 |
|  |  |  |  |  | >8-10 | 1.21 (0.85, 1.72) | 0.29 |
|  |  |  |  |  | >10 | 1.32 (0.72, 2.44) | 0.37 |
| Model 2 ^b^ |  | 1.04 (0.98, 1.10) | 0.23 |  | ≤5 | 1.00 (Reference) |  |
| (9,624) |  |  |  |  | >5-8 | 1.20 (0.86, 1.68) | 0.29 |
|  |  |  |  |  | >8-10 | 1.26 (0.88, 1.80) | 0.21 |
|  |  |  |  |  | >10 | 1.39 (0.75, 2.59) | 0.29 |
| **APOE ε4 carriers** | |  |  |  |  |  |  |
| Unadjusted |  | 1.04 (0.97, 1.10) | 0.27 |  | ≤5 | 1.00 (Reference) |  |
| (3,331) |  |  |  |  | >5-8 | 0.96 (0.69, 1.35) | 0.82 |
|  |  |  |  |  | >8-10 | 1.00 (0.69, 1.44) | 1.00 |
|  |  |  |  |  | >10 | 1.36 (0.75, 2.48) | 0.31 |
| Model 1 ^a^ |  | 1.05 (0.98, 1.12) | 0.15 |  | ≤5 | 1.00 (Reference) |  |
| (3,331) |  |  |  |  | >5-8 | 1.01 (0.72, 1.42) | 0.95 |
|  |  |  |  |  | >8-10 | 1.06 (0.73, 1.53) | 0.76 |
|  |  |  |  |  | >10 | 1.66 (0.91, 3.03) | 0.10 |
| Model 2 ^b^ |  | 1.03 (0.97, 1.10) | 0.33 |  | ≤5 | 1.00 (Reference) |  |
| (3,306) |  |  |  |  | >5-8 | 0.98 (0.70, 1.38) | 0.92 |
|  |  |  |  |  | >8-10 | 1.00 (0.69, 1.46) | 0.99 |
|  |  |  |  |  | >10 | 1.58 (0.86, 2.91) | 0.14 |

^a^: Adjusted for age, sex, and education level.

^b^: Adjusted for age, sex, education level, smoking status, alcohol consumption, body mass index (BMI), state of residence, rurality, and socioeconomic status as indicated by Index of Relative Socio-economic Advantage and Disadvantage (IRSAD).

Supplementary Table 5. Cox proportional hazards models for the association between PM2.5 exposure and incident dementia by age groups

| **Models** | **PM2.5 Exposure (Continuous, µg/m^3^)** | | |  | **PM2.5 Exposure (Cut-offs, µg/m^3^)** | | |
| --- | --- | --- | --- | --- | --- | --- | --- |
| **(N of obs)** |  | **HR (95% CI)** | **P-value** |  |  | **HR (95% CI)** | **P-value** |
| **Aged 70-75** |  |  |  |  |  |  |  |
| Unadjusted |  | 1.01 (0.95, 1.07) | 0.83 |  | ≤5 | 1.00 (Reference) |  |
| (9,320) |  |  |  |  | >5-8 | 1.13 (0.80, 1.59) | 0.49 |
|  |  |  |  |  | >8-10 | 1.03 (0.71, 1.49) | 0.88 |
|  |  |  |  |  | >10 | 1.69 (0.99, 2.90) | 0.06 |
| Model 1 ^a^ |  | 1.01 (0.95, 1.07) | 0.81 |  | ≤5 | 1.00 (Reference) |  |
| (9,320) |  |  |  |  | >5-8 | 1.14 (0.81, 1.60) | 0.46 |
|  |  |  |  |  | >8-10 | 1.03 (0.71, 1.49) | 0.87 |
|  |  |  |  |  | >10 | 1.69 (0.98, 2.90) | 0.06 |
| Model 2 ^b^ |  | 1.01 (0.95, 1.07) | 0.80 |  | ≤5 | 1.00 (Reference) |  |
| (9,260) |  |  |  |  | >5-8 | 1.20 (0.84, 1.70) | 0.32 |
|  |  |  |  |  | >8-10 | 1.07 (0.73, 1.56) | 0.74 |
|  |  |  |  |  | >10 | 1.77 (1.03, 3.07) | **0.040** |
| Model 3 ^c^ |  | 1.02 (0.96, 1.09) | 0.53 |  | ≤5 | 1.00 (Reference) |  |
| (7,690) |  |  |  |  | >5-8 | 1.12 (0.76, 1.64) | 0.56 |
|  |  |  |  |  | >8-10 | 1.03 (0.68, 1.56) | 0.89 |
|  |  |  |  |  | >10 | 1.69 (0.93, 3.06) | 0.08 |
| **Aged 75-80** |  | | |  |  | | |
|  |  |  |  |  |  |  |  |
| Unadjusted |  | 1.03 (0.97, 1.10) | 0.36 |  | ≤5 | 1.00 (Reference) |  |
| (4,273) |  |  |  |  | >5-8 | 1.11 (0.78, 1.58) | 0.58 |
|  |  |  |  |  | >8-10 | 1.13 (0.77, 1.66) | 0.53 |
|  |  |  |  |  | >10 | 1.36 (0.63, 2.94) | 0.44 |
| Model 1 ^a^ |  | 1.04 (0.97, 1.11) | 0.31 |  | ≤5 | 1.00 (Reference) |  |
| (4,272) |  |  |  |  | >5-8 | 1.12 (0.79, 1.60) | 0.52 |
|  |  |  |  |  | >8-10 | 1.15 (0.78, 1.69) | 0.47 |
|  |  |  |  |  | >10 | 1.38 (0.64, 2.98) | 0.42 |
| Model 2 ^b^ |  | 1.02 (0.96, 1.09) | 0.55 |  | ≤5 | 1.00 (Reference) |  |
| (4,241) |  |  |  |  | >5-8 | 1.12 (0.79, 1.60) | 0.53 |
|  |  |  |  |  | >8-10 | 1.09 (0.74, 1.61) | 0.66 |
|  |  |  |  |  | >10 | 1.34 (0.62, 2.91) | 0.46 |
| Model 3 ^c^ |  | 1.07 (0.99, 1.15) | 0.09 |  | ≤5 | 1.00 (Reference) |  |
| (3,392) |  |  |  |  | >5-8 | 1.12 (0.75, 1.69) | 0.58 |
|  |  |  |  |  | >8-10 | 1.26 (0.81, 1.96) | 0.30 |
|  |  |  |  |  | >10 | 1.29 (0.53, 3.16) | 0.58 |
|  |  | | |  |  | | |
| **Aged > 80** |  |  |  |  |  |  |  |
| Unadjusted |  | 1.02 (0.95, 1.09) | 0.63 |  | ≤5 | 1.00 (Reference) |  |
| (2,549) |  |  |  |  | >5-8 | 0.91 (0.63, 1.31) | 0.60 |
|  |  |  |  |  | >8-10 | 1.04 (0.70, 1.54) | 0.86 |
|  |  |  |  |  | >10 | 0.91 (0.40, 2.05) | 0.81 |
| Model 1 ^a^ |  | 1.02 (0.95, 1.09) | 0.66 |  | ≤5 | 1.00 (Reference) |  |
| (2,549) |  |  |  |  | >5-8 | 0.91 (0.63, 1.31) | 0.60 |
|  |  |  |  |  | >8-10 | 1.03 (0.69, 1.54) | 0.87 |
|  |  |  |  |  | >10 | 0.90 (0.40, 2.04) | 0.80 |
| Model 2 ^b^ |  | 1.02 (0.94, 1.09) | 0.70 |  | ≤5 | 1.00 (Reference) |  |
| (2,520) |  |  |  |  | >5-8 | 0.86 (0.60, 1.24) | 0.42 |
|  |  |  |  |  | >8-10 | 1.00 (0.67, 1.49) | 1.00 |
|  |  |  |  |  | >10 | 0.89 (0.39, 2.03) | 0.79 |
| Model 3 ^c^ |  | 1.02 (0.94, 1.11) | 0.64 |  | ≤5 | 1.00 (Reference) |  |
| (1,848) |  |  |  |  | >5-8 | 1.06 (0.67, 1.69) | 0.81 |
|  |  |  |  |  | >8-10 | 1.22 (0.74, 2.00) | 0.44 |
|  |  |  |  |  | >10 | 1.14 (0.42, 3.06) | 0.80 |

^a^: Adjusted for age, sex, and education level.

^b^: Adjusted for age, sex, education level, smoking status, alcohol consumption, body mass index (BMI), state of residence, rurality, and socioeconomic status as indicated by Index of Relative Socio-economic Advantage and Disadvantage (IRSAD).

^c^: Adjusted for age, sex, and education level, smoking status, alcohol consumption, body mass index (BMI), state of residence, rurality, and socioeconomic status as indicated by Index of Relative Socio-economic Advantage and Disadvantage (IRSAD), and APOE ε4 genotype.

Supplementary Table 6. Sensitivity analyses using competing risk model: PM2.5 exposure and incident dementia in the overall population

| **Models ^a^** | **PM2.5 Exposure (Continuous, µg/m^3^)** | | |  | **PM2.5 Exposure (Cut-offs, µg/m^3^)** | | |
| --- | --- | --- | --- | --- | --- | --- | --- |
| **(N of obs)** |  | **SHR (95% CI)** | **P-value** |  |  | **SHR (95% CI)** | **P-value** |
| Unadjusted |  | 1.00 (0.96, 1.04) | 0.99 |  | ≤5 | 1.00 (Reference) |  |
| (16,145) |  |  |  |  | >5-8 | 1.03 (0.84, 1.26) | 0.77 |
|  |  |  |  |  | >8-10 | 1.00 (0.79, 1.24) | 0.94 |
|  |  |  |  |  | >10 | 1.17 (0.80, 1.70) | 0.43 |
| Model 1 ^b^ |  | 1.00 (0.97, 1.04) | 0.80 |  | ≤5 | 1.00 (Reference) |  |
| (16,144) |  |  |  |  | >5-8 | 1.04 (0.85, 1.28) | 0.72 |
|  |  |  |  |  | >8-10 | 1.02 (0.82, 1.27) | 0.86 |
|  |  |  |  |  | >10 | 1.21 (0.83, 1.78) | 0.32 |
| Model 2 ^c^ |  | 1.00 (0.96, 1.04) | 0.94 |  | ≤5 | 1.00 (Reference) |  |
| (16,021) |  |  |  |  | >5-8 | 1.04 (0.84, 1.28) | 0.73 |
|  |  |  |  |  | >8-10 | 1.00 (0.80, 1.26) | 0.97 |
|  |  |  |  |  | >10 | 1.23 (0.84, 1.82) | 0.29 |
| Model 3 ^d^ |  | 1.02 (0.98, 1.07) | 0.33 |  | ≤5 | 1.00 (Reference) |  |
| (12,930) |  |  |  |  | >5-8 | 1.09 (0.86, 1.38) | 0.50 |
|  |  |  |  |  | >8-10 | 1.09 (0.84, 1.41) | 0.52 |
|  |  |  |  |  | >10 | 1.33 (0.86, 2.05) | 0.20 |

^a^: All models treated death as a competing event.

^b^: Adjusted for age, sex, and education level.

^c^: Adjusted for age, sex, education level, smoking status, alcohol consumption, body mass index (BMI), state of residence, rurality, and socioeconomic status as indicated by Index of Relative Socio-economic Advantage and Disadvantage (IRSAD).

^d^: Adjusted for age, sex, and education level, smoking status, alcohol consumption, body mass index (BMI), state of residence, rurality, and socioeconomic status as indicated by Index of Relative Socio-economic Advantage and Disadvantage (IRSAD), and APOE ε4 genotype.

Supplementary Table 7. Sensitivity analyses using competing risk model: PM2.5 exposure and incident dementia in females

| **Models ^a^** | **PM2.5 Exposure (Continuous, µg/m^3^)** | | |  | **PM2.5 Exposure (Cut-offs, µg/m^3^)** | | |
| --- | --- | --- | --- | --- | --- | --- | --- |
| **(N of obs)** |  | **SHR (95% CI)** | **P-value** |  |  | **SHR (95% CI)** | **P-value** |
| Unadjusted |  | 1.03 (0.98, 1.09) | 0.28 |  | ≤5 | 1.00 (Reference) |  |
| (8,858) |  |  |  |  | >5-8 | 1.00 (0.75, 1.34) | 0.99 |
|  |  |  |  |  | >8-10 | 0.97 (0.71, 1.33) | 0.87 |
|  |  |  |  |  | >10 | 1.81 (1.13, 2.87) | **0.013** |
| Model 1 ^b^ |  | 1.04 (0.98, 1.10) | 0.16 |  | ≤5 | 1.00 (Reference) |  |
| (8,857) |  |  |  |  | >5-8 | 1.03 (0.77, 1.37) | 0.85 |
|  |  |  |  |  | >8-10 | 1.03 (0.76, 1.41) | 0.84 |
|  |  |  |  |  | >10 | 1.95 (1.22, 3.13) | **0.005** |
| Model 2 ^c^ |  | 1.03 (0.98, 1.09) | 0.25 |  | ≤5 | 1.00 (Reference) |  |
| (8,784) |  |  |  |  | >5-8 | 1.04 (0.77, 1.39) | 0.81 |
|  |  |  |  |  | >8-10 | 1.00 (0.73, 1.37) | 0.99 |
|  |  |  |  |  | >10 | 1.98 (1.23, 3.20) | **0.003** |
| Model 3 ^d^ |  | 1.06 (0.99, 1.12) | 0.08 |  | ≤5 | 1.00 (Reference) |  |
| (6,949) |  |  |  |  | >5-8 | 1.22 (0.86, 1.74) | 0.26 |
|  |  |  |  |  | >8-10 | 1.18 (0.81, 1.72) | 0.40 |
|  |  |  |  |  | >10 | 2.47 (1.41, 4.32) | **0.003** |

^a^: All models treated death as a competing event.

^b^: Adjusted for age, sex, and education level.

^c^: Adjusted for age, sex, education level, smoking status, alcohol consumption, body mass index (BMI), state of residence, rurality, and socioeconomic status as indicated by Index of Relative Socio-economic Advantage and Disadvantage (IRSAD).

^d^: Adjusted for age, sex, and education level, smoking status, alcohol consumption, body mass index (BMI), state of residence, rurality, and socioeconomic status as indicated by Index of Relative Socio-economic Advantage and Disadvantage (IRSAD), and APOE ε4 genotype.

Supplementary Table 8. Sensitivity analyses using competing risk model: PM2.5 exposure and incident dementia in males

| **Models ^a^** | **PM2.5 Exposure (Continuous, µg/m^3^)** | | |  | **PM2.5 Exposure (Cut-offs, µg/m^3^)** | | |
| --- | --- | --- | --- | --- | --- | --- | --- |
| **(N of obs)** |  | **SHR (95% CI)** | **P-value** |  |  | **SHR (95% CI)** | **P-value** |
| Unadjusted |  | 0.97 (0.92, 1.02) | 0.22 |  | ≤5 | 1.00 (Reference) |  |
| (7,287) |  |  |  |  | >5-8 | 1.07 (0.80, 1.43) | 0.66 |
|  |  |  |  |  | >8-10 | 1.01 (0.74, 1.38) | 0.97 |
|  |  |  |  |  | >10 | 0.56 (0.28, 1.14) | 0.11 |
| Model 1 ^b^ |  | 0.97 (0.92, 1.02) | 0.24 |  | ≤5 | 1.00 (Reference) |  |
| (7,287) |  |  |  |  | >5-8 | 1.06 (0.79, 1.41) | 0.72 |
|  |  |  |  |  | >8-10 | 1.01 (0.74, 1.38) | 0.94 |
|  |  |  |  |  | >10 | 0.56 (0.28, 1.15) | 0.12 |
| Model 2 ^c^ |  | 0.97 (0.92, 1.02) | 0.26 |  | ≤5 | 1.00 (Reference) |  |
| (7,237) |  |  |  |  | >5-8 | 1.06 (0.78, 1.42) | 0.72 |
|  |  |  |  |  | >8-10 | 1.03 (0.74, 1.41) | 0.88 |
|  |  |  |  |  | >10 | 0.57 (0.28, 1.16) | 0.12 |
| Model 3 ^d^ |  | 0.99 (0.93, 1.05) | 0.69 |  | ≤5 | 1.00 (Reference) |  |
| (5,981) |  |  |  |  | >5-8 | 0.98 (0.71, 1.36) | 0.91 |
|  |  |  |  |  | >8-10 | 1.02 (0.72, 1.45) | 0.92 |
|  |  |  |  |  | >10 | 0.63 (0.30, 1.30) | 0.21 |

^a^: All models treated death as a competing event.

^b^: Adjusted for age, sex, and education level.

^c^: Adjusted for age, sex, education level, smoking status, alcohol consumption, body mass index (BMI), state of residence, rurality, and socioeconomic status as indicated by Index of Relative Socio-economic Advantage and Disadvantage (IRSAD).

^d^: Adjusted for age, sex, and education level, smoking status, alcohol consumption, body mass index (BMI), state of residence, rurality, and socioeconomic status as indicated by Index of Relative Socio-economic Advantage and Disadvantage (IRSAD), and APOE ε4 genotype.

Supplementary Table 9. Sensitivity analyses with Cox proportional hazards models for the association between 3-year mean PM2.5 exposure and incident dementia in the overall population

| **Models** | **PM2.5 Exposure (Continuous, µg/m^3^)** | | |  | **PM2.5 Exposure (Cut-offs, µg/m^3^)** | | |
| --- | --- | --- | --- | --- | --- | --- | --- |
| **(N of obs)** |  | **HR (95% CI)** | **P-value** |  |  | **HR (95% CI)** | **P-value** |
| Unadjusted |  | 1.01 (0.98, 1.04) | 0.72 |  | ≤5 | 1.00 (Reference) |  |
| (16,145) |  |  |  |  | >5-8 | 1.05 (0.85, 1.29) | 0.65 |
|  |  |  |  |  | >8-10 | 1.04 (0.84, 1.29) | 0.72 |
|  |  |  |  |  | >10 | 1.04 (0.79, 1.37) | 0.76 |
| Model 1 ^a^ |  | 1.00 (0.97, 1.04) | 0.76 |  | ≤5 | 1.00 (Reference) |  |
| (16,144) |  |  |  |  | >5-8 | 1.07 (0.87, 1.32) | 0.52 |
|  |  |  |  |  | >8-10 | 1.07 (0.86, 1.33) | 0.54 |
|  |  |  |  |  | >10 | 1.01 (0.77, 1.33) | 0.92 |
| Model 2 ^b^ |  | 1.01 (0.98, 1.04) | 0.69 |  | ≤5 | 1.00 (Reference) |  |
| (16,021) |  |  |  |  | >5-8 | 1.07 (0.87, 1.32) | 0.52 |
|  |  |  |  |  | >8-10 | 1.08 (0.87, 1.35) | 0.47 |
|  |  |  |  |  | >10 | 1.02 (0.78, 1.34) | 0.88 |
| Model 3 ^c^ |  | 1.02 (0.99, 1.06) | 0.19 |  | ≤5 | 1.00 (Reference) |  |
| (12,930) |  |  |  |  | >5-8 | 1.11 (0.88, 1.42) | 0.38 |
|  |  |  |  |  | >8-10 | 1.19 (0.93, 1.53) | 0.18 |
|  |  |  |  |  | >10 | 1.17 (0.86, 1.59) | 0.31 |

^a^: Adjusted for age, sex, and education level.

^b^: Adjusted for age, sex, education level, smoking status, alcohol consumption, body mass index (BMI), state of residence, rurality, and socioeconomic status as indicated by Index of Relative Socio-economic Advantage and Disadvantage (IRSAD).

^c^: Adjusted for age, sex, and education level, smoking status, alcohol consumption, body mass index (BMI), state of residence, rurality, and socioeconomic status as indicated by Index of Relative Socio-economic Advantage and Disadvantage (IRSAD), and APOE ε4 genotype.

Supplementary Table 10. Sensitivity analyses with Cox proportional hazards models for the association between 3-year mean PM2.5 exposure and incident dementia in females

| **Models** | **PM2.5 Exposure (Continuous, µg/m^3^)** | | |  | **PM2.5 Exposure (Cut-offs, µg/m^3^)** | | |
| --- | --- | --- | --- | --- | --- | --- | --- |
| **(N of obs)** |  | **HR (95% CI)** | **P-value** |  |  | **HR (95% CI)** | **P-value** |
| Unadjusted |  | 1.01 (0.97, 1.05) | 0.76 |  | ≤5 | 1.00 (Reference) |  |
| (8,856) |  |  |  |  | >5-8 | 1.09 (0.81, 1.47) | 0.56 |
|  |  |  |  |  | >8-10 | 0.99 (0.73, 1.35) | 0.95 |
|  |  |  |  |  | >10 | 1.15 (0.79, 1.67) | 0.48 |
| Model 1 ^a^ |  | 1.01 (0.97, 1.05) | 0.74 |  | ≤5 | 1.00 (Reference) |  |
| (8,855) |  |  |  |  | >5-8 | 1.13 (0.84, 1.52) | 0.40 |
|  |  |  |  |  | >8-10 | 1.03 (0.75, 1.41) | 0.85 |
|  |  |  |  |  | >10 | 1.13 (0.77, 1.65) | 0.53 |
| Model 2 ^b^ |  | 1.01 (0.97, 1.06) | 0.54 |  | ≤5 | 1.00 (Reference) |  |
| (8,784) |  |  |  |  | >5-8 | 1.15 (0.85, 1.55) | 0.35 |
|  |  |  |  |  | >8-10 | 1.07 (0.78, 1.46) | 0.69 |
|  |  |  |  |  | >10 | 1.18 (0.81, 1.73) | 0.40 |
| Model 3 ^c^ |  | 1.02 (0.97, 1.07) | 0.42 |  | ≤5 | 1.00 (Reference) |  |
| (6,949) |  |  |  |  | >5-8 | 1.38 (0.96, 1.97) | 0.082 |
|  |  |  |  |  | >8-10 | 1.24 (0.85, 1.81) | 0.26 |
|  |  |  |  |  | >10 | 1.38 (0.88, 2.15) | 0.16 |

^a^: Adjusted for age, sex, and education level.

^b^: Adjusted for age, sex, education level, smoking status, alcohol consumption, body mass index (BMI), state of residence, rurality, and socioeconomic status as indicated by Index of Relative Socio-economic Advantage and Disadvantage (IRSAD).

^c^: Adjusted for age, sex, and education level, smoking status, alcohol consumption, body mass index (BMI), state of residence, rurality, and socioeconomic status as indicated by Index of Relative Socio-economic Advantage and Disadvantage (IRSAD), and APOE ε4 genotype.

Supplementary Table 11. Sensitivity analyses with Cox proportional hazards models for the association between 3-year mean PM2.5 exposure and incident dementia in males

| **Models** | **PM2.5 Exposure (Continuous, µg/m^3^)** | | |  | **PM2.5 Exposure (Cut-offs, µg/m^3^)** | | |
| --- | --- | --- | --- | --- | --- | --- | --- |
| **(N of obs)** |  | **HR (95% CI)** | **P-value** |  |  | **HR (95% CI)** | **P-value** |
| Unadjusted |  | 1.00 (0.96, 1.05) | 0.83 |  | ≤5 | 1.00 (Reference) |  |
| (7,286) |  |  |  |  | >5-8 | 1.01 (0.76, 1.36) | 0.93 |
|  |  |  |  |  | >8-10 | 1.10 (0.81, 1.50) | 0.53 |
|  |  |  |  |  | >10 | 0.94 (0.64, 1.39) | 0.76 |
| Model 1 ^a^ |  | 1.00 (0.96, 1.05) | 0.94 |  | ≤5 | 1.00 (Reference) |  |
| (7,286) |  |  |  |  | >5-8 | 1.01 (0.75, 1.35) | 0.96 |
|  |  |  |  |  | >8-10 | 1.12 (0.82, 1.51) | 0.48 |
|  |  |  |  |  | >10 | 0.90 (0.61, 1.33) | 0.60 |
| Model 2 ^b^ |  | 1.00 (0.96, 1.05) | 0.98 |  | ≤5 | 1.00 (Reference) |  |
| (7,237) |  |  |  |  | >5-8 | 1.02 (0.75, 1.37) | 0.92 |
|  |  |  |  |  | >8-10 | 1.12 (0.82, 1.52) | 0.48 |
|  |  |  |  |  | >10 | 0.90 (0.61, 1.34) | 0.61 |
| Model 3 ^c^ |  | 1.03 (0.98, 1.08) | 0.28 |  | ≤5 | 1.00 (Reference) |  |
| (5,981) |  |  |  |  | >5-8 | 0.92 (0.66, 1.28) | 0.62 |
|  |  |  |  |  | >8-10 | 1.15 (0.82, 1.61) | 0.41 |
|  |  |  |  |  | >10 | 1.04 (0.68, 1.60) | 0.86 |

^a^: Adjusted for age, sex, and education level.

^b^: Adjusted for age, sex, education level, smoking status, alcohol consumption, body mass index (BMI), state of residence, rurality, and socioeconomic status as indicated by Index of Relative Socio-economic Advantage and Disadvantage (IRSAD).

^c^: Adjusted for age, sex, and education level, smoking status, alcohol consumption, body mass index (BMI), state of residence, rurality, and socioeconomic status as indicated by Index of Relative Socio-economic Advantage and Disadvantage (IRSAD), and APOE ε4 genotype.

Supplementary Table 12. Sensitivity analyses using shorter follow-up periods: Cox proportional hazards models for the association between 1-year mean PM2.5 exposure and incident dementia in the overall population

| **Models** | **PM2.5 Exposure (Continuous, µg/m^3^)** | | |  | **PM2.5 Exposure (Cut-offs, µg/m^3^)** | | |
| --- | --- | --- | --- | --- | --- | --- | --- |
| **(N of obs)** |  | **HR (95% CI)** | **P-value** |  |  | **HR (95% CI)** | **P-value** |
| Unadjusted |  | 1.04 (0.99, 1.11) | 0.22 |  | ≤5 | 1.00 (Reference) |  |
| (16,145) |  |  |  |  | >5-8 | 1.18(0.83, 1.67) | 0.37 |
|  |  |  |  |  | >8-10 | 1.22 (0.83, 1.80) | 0.31 |
|  |  |  |  |  | >10 | 0.96 (0.40, 2.28) | 0.92 |
| Model 1 ^a^ |  | 1.04 (0.98, 1.11) | 0.20 |  | ≤5 | 1.00 (Reference) |  |
| (16,144) |  |  |  |  | >5-8 | 1.17 (0.82, 1.66) | 0.40 |
|  |  |  |  |  | >8-10 | 1.24 (0.84, 1.82) | 0.28 |
|  |  |  |  |  | >10 | 1.04 (0.44, 2.49) | 0.93 |
| Model 2 ^b^ |  | 1.04 (0.98, 1.12) | 0.19 |  | ≤5 | 1.00 (Reference) |  |
| (16,021) |  |  |  |  | >5-8 | 1.16 (0.82, 1.66) | 0.40 |
|  |  |  |  |  | >8-10 | 1.22 (0.84, 1.82) | 0.28 |
|  |  |  |  |  | >10 | 1.05 (0.45, 2.60) | 0.85 |
| Model 3 ^c^ |  | 1.08 (0.99, 1.17) | 0.072 |  | ≤5 | 1.00 (Reference) |  |
| (12,930) |  |  |  |  | >5-8 | 1.30 (0.83, 2.03) | 0.26 |
|  |  |  |  |  | >8-10 | 1.49 (0.92, 2.41) | 0.11 |
|  |  |  |  |  | >10 | 0.95 (0.28, 3.21) | 0.94 |

^a^: Adjusted for age, sex, and education level.

^b^: Adjusted for age, sex, education level, smoking status, alcohol consumption, body mass index (BMI), state of residence, rurality, and socioeconomic status as indicated by Index of Relative Socio-economic Advantage and Disadvantage (IRSAD).

^c^: Adjusted for age, sex, and education level, smoking status, alcohol consumption, body mass index (BMI), state of residence, rurality, and socioeconomic status as indicated by Index of Relative Socio-economic Advantage and Disadvantage (IRSAD), and APOE ε4 genotype.

Supplementary Table 13. Sensitivity analyses using shorter follow-up periods: Cox proportional hazards models for the association between 1-year mean PM2.5 exposure and incident dementia in females

| **Models** | **PM2.5 Exposure (Continuous, µg/m^3^)** | | |  | **PM2.5 Exposure (Cut-offs, µg/m^3^)** | | |
| --- | --- | --- | --- | --- | --- | --- | --- |
| **(N of obs)** |  | **HR (95% CI)** | **P-value** |  |  | **HR (95% CI)** | **P-value** |
| Unadjusted |  | 1.07 (0.97, 1.17) | 0.17 |  | ≤5 | 1.00 (Reference) |  |
| (8,858) |  |  |  |  | >5-8 | 0.94 (0.59, 1.49) | 0.79 |
|  |  |  |  |  | >8-10 | 1.05 (0.63, 1.75) | 0.85 |
|  |  |  |  |  | >10 | 1.74 (0.70, 4.35) | 0.24 |
| Model 1 ^a^ |  | 1.08 (0.98, 1.18) | 0.12 |  | ≤5 | 1.00 (Reference) |  |
| (8,857) |  |  |  |  | >5-8 | 0.97 (0.61, 1.54) | 0.88 |
|  |  |  |  |  | >8-10 | 1.12 (0.67, 1.87) | 0.66 |
|  |  |  |  |  | >10 | 1.97 (0.79, 4.94) | 0.15 |
| Model 2 ^b^ |  | 1.08 (0.98, 1.18) | 0.12 |  | ≤5 | 1.00 (Reference) |  |
| (8,784) |  |  |  |  | >5-8 | 0.97 (0.61, 1.55) | 0.91 |
|  |  |  |  |  | >8-10 | 1.11 (0.66, 1.86) | 0.69 |
|  |  |  |  |  | >10 | 2.15 (0.86, 5.41) | 0.10 |
| Model 3 ^c^ |  | 1.13 (1.01, 1.27) | **0.037** |  | ≤5 | 1.00 (Reference) |  |
| (6,949) |  |  |  |  | >5-8 | 1.32 (0.69, 2.53) | 0.40 |
|  |  |  |  |  | >8-10 | 1.59 (0.79, 3.20) | 0.19 |
|  |  |  |  |  | >10 | 2.52 (0.68, 9.30) | 0.16 |

^a^: Adjusted for age, sex, and education level.

^b^: Adjusted for age, sex, education level, smoking status, alcohol consumption, body mass index (BMI), state of residence, rurality, and socioeconomic status as indicated by Index of Relative Socio-economic Advantage and Disadvantage (IRSAD).

^c^: Adjusted for age, sex, and education level, smoking status, alcohol consumption, body mass index (BMI), state of residence, rurality, and socioeconomic status as indicated by Index of Relative Socio-economic Advantage and Disadvantage (IRSAD), and APOE ε4 genotype.

Supplementary Table 14. Sensitivity analyses using shorter follow-up periods: Cox proportional hazards models for the association between 1-year mean PM2.5 exposure and incident dementia in males

| **Models** | **PM2.5 Exposure (Continuous, µg/m^3^)** | | |  | **PM2.5 Exposure (Cut-offs, µg/m^3^)** | | |
| --- | --- | --- | --- | --- | --- | --- | --- |
| **(N of obs)** |  | **HR (95% CI)** | **P-value** |  |  | **HR (95% CI)** | **P-value** |
| Unadjusted |  | 1.02 (0.93, 1.11) | 0.72 |  | ≤5 | 1.00 (Reference) |  |
| (7,287) |  |  |  |  | >5-8 | 1.54 (0.89, 2.65) | 0.12 |
|  |  |  |  |  | >8-10 | 1.47 (0.82, 2.65) | 0.20 |
|  |  |  |  |  | >10 | - | - |
| Model 1 ^a^ |  | 1.01 (0.92, 1.11) | 0.82 |  | ≤5 | 1.00 (Reference) |  |
| (7,287) |  |  |  |  | >5-8 | 1.46 (0.85, 2.52) | 0.17 |
|  |  |  |  |  | >8-10 | 1.42 (0.79, 2.56) | 0.24 |
|  |  |  |  |  | >10 | - | - |
| Model 2 ^b^ |  | 1.02 (0.93, 1.12) | 0.70 |  | ≤5 | 1.00 (Reference) |  |
| (7,237) |  |  |  |  | >5-8 | 1.49 (0.86, 2.58) | 0.15 |
|  |  |  |  |  | >8-10 | 1.49 (0.83, 2.70) | 0.18 |
|  |  |  |  |  | >10 | - | - |
| Model 3 ^c^ |  | 1.03 (0.93, 1.15) | 0.57 |  | ≤5 | 1.00 (Reference) |  |
| (5,981) |  |  |  |  | >5-8 | 1.33 (0.71, 2.47) | 0.37 |
|  |  |  |  |  | >8-10 | 1.45 (0.74, 2.84) | 0.27 |
|  |  |  |  |  | >10 | - | - |

^a^: Adjusted for age, sex, and education level.

^b^: Adjusted for age, sex, education level, smoking status, alcohol consumption, body mass index (BMI), state of residence, rurality, and socioeconomic status as indicated by Index of Relative Socio-economic Advantage and Disadvantage (IRSAD).

^c^: Adjusted for age, sex, and education level, smoking status, alcohol consumption, body mass index (BMI), state of residence, rurality, and socioeconomic status as indicated by Index of Relative Socio-economic Advantage and Disadvantage (IRSAD), and APOE ε4 genotype.
